# SG-APSIC1042: Cutaneous cryptococcosis in patient with advanced HIV disease: Is it possible to give antifungal monotherapy?

**DOI:** 10.1017/ash.2023.34

**Published:** 2023-03-16

**Authors:** Visakha Revana Irawan, Agung Nugroho, Pearla Lasut

**Affiliations:** Internal Medicine Department, Faculty of Medicine, Sam Ratulangi University, Prof. Dr. R. D. Kandou General Hospital, Manado, Indonesia; Division of Tropical Medicine and Infectious Diseases, Department of Internal Medicine, Faculty of Medicine, Sam Ratulangi University, Prof. Dr. R. D. Kandou General Hospital, Manado, Indonesia; Division of Hematology and Medical Oncology, Department of Internal Medicine, Faculty of Medicine, Sam Ratulangi University, Prof. Dr. R. D. Kandou General Hospital, Manado, Indonesia

## Abstract

**Objectives:** *Cryptococcus* infection is one of the major human immunodeficiency virus (HIV)–related opportunistic infections, and the CD4 count falls below 100 per µL. Primary treatment for HIV-associated cutaneous cryptococcosis is amphotericin B (AmB) plus flucytosine. **Methods:** We present the case of a man with advanced HIV disease who developed whole-body cutaneous lesions yet improved with high-dose fluconazole alone. **Results:** A 33-year-old Asian man with a medical history of pulmonary tuberculosis and cryptococcal meningitis with complete treatment, injection drug use, and HIV infection with default of antiretroviral treatments (ART) 3 years earlier, presented to the emergency department with fever, oral thrush, and 30-pound weight loss over 6 weeks. He also had plaques, multiple hard papulonodules with central ulceration, and macular skin lesions all over his body of varying size. Blood cultures were negative for bacteria growth, but fungal microscopy of the blood culture showed unspecific hypha. Histopathology examination of the skin biopsy showed a classic “soap bubble” appearance, which is associated with *Cryptococcus* infection. Laboratory values revealed anemia (8.6 g/dL), leukopenia (2.9 ×10^9^/L), lymphopenia (58/µL), and thrombocytopenia (145 ×10^9^/L). The CD4 cell count was 18/µL, and the serum viral load was 638.665 copies/mL. Lumbar puncture could not be performed due to patient refusal. Treatment with high-dose fluconazole (1,200 mg) for 3 months was initiated and is planned to continue with consolidation and maintenance dose. ART was administered 4 weeks after starting antifungal therapy. His fever resolved and slow regression of the skin lesions occurred after treatment was given. **Conclusions:** Cutaneous cryptococcosis was assessed by biopsy of the cutaneous lesion, which is essential to confirming the diagnosis. In the case of cryptococcosis, skin infection may indicate a further progression of advanced HIV disease. In HIV-infected patients with Cryptococcus findings in any part of the body, a lumbar puncture should be considered to rule out central nervous system infection. Although neither AmB nor flucytosine was given due to unavailability in this area, the patient improved. In resource-limited settings, high-dose fluconazole alone may be useful as an alternative treatment, although it is also very challenging.

